# Feature Representations for Neuromorphic Audio Spike Streams

**DOI:** 10.3389/fnins.2018.00023

**Published:** 2018-02-09

**Authors:** Jithendar Anumula, Daniel Neil, Tobi Delbruck, Shih-Chii Liu

**Affiliations:** Institute of Neuroinformatics, University of Zurich and ETH Zurich, Zurich, Switzerland

**Keywords:** dynamic audio sensor, spike feature generation, exponential kernels, recurrent neural network, audio word classification

## Abstract

Event-driven neuromorphic spiking sensors such as the silicon retina and the silicon cochlea encode the external sensory stimuli as asynchronous streams of spikes across different channels or pixels. Combining state-of-art deep neural networks with the asynchronous outputs of these sensors has produced encouraging results on some datasets but remains challenging. While the lack of effective spiking networks to process the spike streams is one reason, the other reason is that the pre-processing methods required to convert the spike streams to frame-based features needed for the deep networks still require further investigation. This work investigates the effectiveness of synchronous and asynchronous frame-based features generated using spike count and constant event binning in combination with the use of a recurrent neural network for solving a classification task using N-TIDIGITS18 dataset. This spike-based dataset consists of recordings from the Dynamic Audio Sensor, a spiking silicon cochlea sensor, in response to the TIDIGITS audio dataset. We also propose a new pre-processing method which applies an exponential kernel on the output cochlea spikes so that the interspike timing information is better preserved. The results from the N-TIDIGITS18 dataset show that the exponential features perform better than the spike count features, with over 91% accuracy on the digit classification task. This accuracy corresponds to an improvement of at least 2.5% over the use of spike count features, establishing a new state of the art for this dataset.

## 1. Introduction

The event processing methods for the asynchronous spikes of event-based sensors such as the Dynamic Vision Sensor (DVS) (Lichtsteiner et al., 2008; Berner et al., 2013; Posch et al., 2014; Yang et al., 2015) and the Dynamic Audio Sensor (DAS) (Liu et al., 2014; Yang et al., 2016) fall roughly into two categories: either by the use of neural network methods or machine learning algorithms. These methods have been primarily developed for event-based vision sensors and with the availability of DVS datasets (Orchard et al., 2015; Serrano-Gotarredona and Linares-Barranco, 2015; Barranco et al., 2016), performances of these methods can be compared.

In recent years, the field of deep learning has seen major developments leading to networks that achieve state-of-art performance on complex tasks such as speech recognition and visual object recognition (Schmidhuber, 2014; LeCun et al., 2015). With event-based sensors finding increasing relevance in event-driven artificial sensory or cognitive systems, there has been a new effort in interfacing these sensors with these powerful machine learning networks. However, deep learning frameworks typically use frame-based data. To interface the output of the event-based sensors to the deep network, there are two alternative methods. The first method is to present the spikes to spiking deep networks as has been reported (Farabet et al., 2012; Pérez-Carrasco et al., 2013; Zhao et al., 2015; Esser et al., 2016; Amir et al., 2017). By using conversion methods that convert pre-trained standard deep networks into equivalent-accurate spiking networks (Diehl et al., 2015; Rueckauer et al., 2017) or by using the training methods from deep learning on networks that capture the underlying parameters of the spiking neuron (O'Connor et al., 2013; Stromatias et al., 2015), we are starting to see spiking deep networks that can be competitive with the standard deep networks.

Another method is to create either synchronous or asynchronous feature frames from the spikes before presentation to the time-stepped deep networks. This method has seen success in the field of neuromorphic vision primarily, as pre-processing methods produce frames from event-driven sensor data to use as inputs to deep networks for classification tasks (Moeys et al., 2016; Neil and Liu, 2016; Lungu et al., 2017). Although these pre-processing methods are outperformed on standard classification tasks by the methods using the traditional frame based sensors, they can help reduce computation by using the data driven nature of the sensors and processing the networks only when the sensor produces events.

This work aims to methodically examine existing and novel spike pre-processing methods for processing the output of the DAS for use with deep networks and machine learning algorithms, in particular for real-time applications. We consider two existing feature extraction methods that generate feature frames using spike counts within a fixed time bin and constant spike count (event) bins respectively. We also propose a new pre-processing method that generates feature frames by applying an exponential kernel to each event. We compare the performances of the different pre-processing methods by combining them with deep learning recurrent neural networks which include gated units (Chung et al., 2014; Neil et al., 2016) and testing the networks on two audio classification tasks (isolated recordings and connected streams) using a recorded audio spike dataset called N-TIDIGITS18. This dataset consists of spike recordings from a Dynamic Audio Sensor in response to the TIDIGITS (Leonard and Doddington, 1993) audio dataset.

## 2. Methods

This section presents a description of the hardware cochlea sensors, details the feature generation methods, including the proposed exponential feature generation method and briefly describes the deep network architectures used in this study.

### 2.1. Dynamic audio sensor

**Figure 1:**
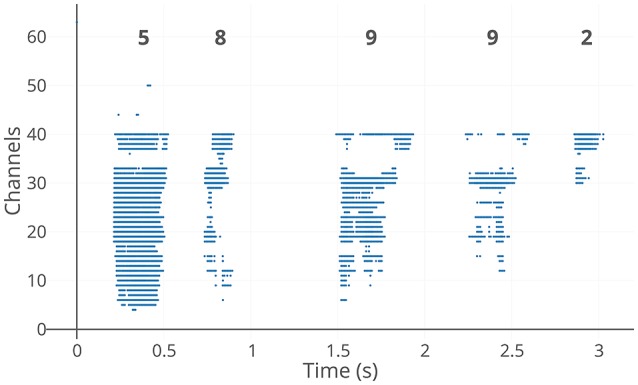
CochleaAMS1c spike output example. The y-axis indicates the 64 frequency channels of the sensor with lower frequency channels at the top. The spikes are in response to the spoken digit sequence “5-8-9-9-2” from the speaker “IM” in the TIDIGITS dataset. The five digits in the sequence can be clearly seen to be apart with significant gaps between them in the encoded sample above. This example also demonstrates the data driven nature of the sensor where it outputs events only when there's a stimulus in the environment.

The Dynamic Audio Sensor is a binaural silicon cochlea system, with each ear connected to a set of 64 bandpass filters whose center frequencies are logarithmically distributed from approximately 50 Hz to 20 kHz. The events are then asynchronously generated from each of the filters. A silicon cochlea sensor using half wave rectification for the generation of events is the CochleaAMS1b (Chan et al., 2007) and the CochleaAMS1c (Liu et al., 2014), while a cochlea sensor using asynchronous delta modulation for the generation of events is the CochLP (Yang et al., 2016). The CochleaAMS1c sensor is an improved design of the CochleaAMS1b. Each channel of the CochleaAMS1b and CochleaAMS1c has four neurons and each neuron implements a different threshold level for spike generation. In many of the experiments, only the events from a single neuron of one ear are used. An example output for the CochleaAMS1c is shown in Figure 1. The methods evaluated in this work were carried out on recordings from the CochleaAMS1b and CochleaAMS1c, while they will be evaluated on CochLP in the future.

### 2.2. Feature extraction methods

The event data from the cochlea sensors can be converted to frame-based features through multiple methods. One commonly used feature type is the Spike Count (SC) feature (Zai et al., 2015; Anumula et al., 2017), that is generated by the creation of a histogram across the frequency channels of the events within a time window. In the case of the DAS, the feature vector for each time frame is, at maximum, a 64-length vector where each element consists of the number of events in that frequency channel. The two main variants of SC features are time-binned and event-binned features. Their formulation is described below.

#### 2.2.1. Raw spikes

An audio event stream can be mathematically represented as

(1) $$\begin{array}{l} e_{i}=\left[t_{i},\;f_{i}\right],i\in \mathbb{N} \end{array}$$

where *e*_*i*_ is the *i*th event from the frequency channel *f*_*i*_ in the event stream at time *t*_*i*_. The *f*_*i*_ can range between 1 and *N*_*c*_ where *N*_*c*_ is the number of frequency channels in the sensor. Also note that the events are time ordered, i.e., for *i* < *j*, *t*_*i*_ ≤ *t*_*j*_. These raw spike information can be processed directly as a sequence by the recurrent networks. Such a method is not usually feasible though because of the inability of the standard recurrent networks to process longer sequences, but they can be efficiently processed through the Phased LSTM, a recently introduced gated recurrent network architecture (Neil et al., 2016).

#### 2.2.2. Time-binned spike count features

For the generation of time binned Spike Count features, the frame duration for generating the feature is of fixed time length. Time-binned SC features have been used for the speaker identification task using spike recordings generated from the TIMIT dataset (Liu et al., 2010; Li et al., 2012), the YOHO dataset (Chakrabartty and Liu, 2010), and real-world DAS recordings (Anumula et al., 2017).

The time-binned SC features *F*^*tb*^ for a time window length of *T*_*l*_ are defined as follows:

(2) $$\begin{array}{l} F_{j}^{tb}\left(f\right)=\text{card}\left(\left\{e_{i}|T_{l}\cdot \left(j-1\right)\leq t_{i}<T_{l}\cdot j,\;f_{i}=f\right\}\right) \end{array}$$

where $F_{j}^{tb}$ is the *j*th frame of the features, **card**() is the cardinality of a set, · is the standard multiplication operator, and *f* is the position of the frequency channel.

**Figure 2:**
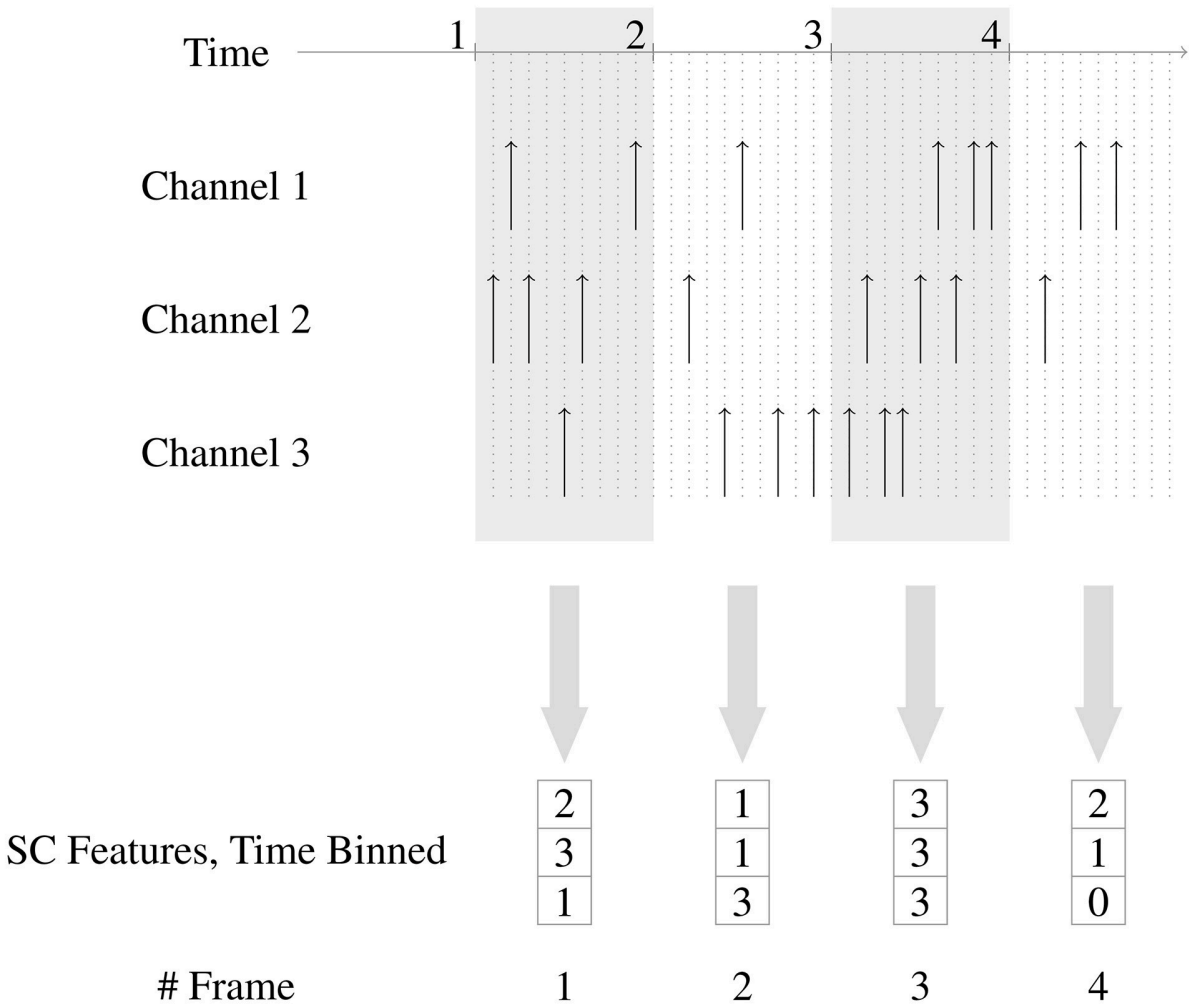
Generation of time-binned Spike Count features. Three channels are shown in this example. The fixed length time windows used for binning the events are non overlapping and of unit time length. In frame 2, there is 1 event in channel 1, 1 event in channel 2 and 3 events in channel 3, and hence the corresponding feature is (1, 1, 3).

Figure 2 shows how the time-binned SC features are generated from the spikes.

#### 2.2.3. Event-binned spike count features

Event-binned SC features consist of frames in which there are a fixed number of events. Unlike time-binned spike count features, event binning is a data driven approach and eliminates the need for input normalization. These features have been used for both the DVS and the DAS. In the robot predator-prey scenario in Moeys et al. (2016), the DVS retina data is integrated into 36 × 36 frames as 2D histograms obtained by integrating 5,000 events in 200 possible gray level values. Since the DVS frames are sparse, active DVS frame pixels accumulate about 50 events. Constant-event frames from the spiking TIMIT dataset have also been used together with a Support Vector Machine Classifier in a speaker identification task (Li et al., 2012).

The event-binned spike count features *F*^*eb*^ are defined as follows. The *j*th frame is given by

(3) $$\begin{array}{l} F_{j}^{eb}\left(f\right)=\text{card}\left(\left\{e_{i}|E\cdot \left(j-1\right)\leq i<E\cdot j,\;f_{i}=f\right\}\right) \end{array}$$

where **card**() is the cardinality of a set, · is the standard multiplication operation, *f* is the position of the frequency channel and *E* is the number of events binned into a single frame.

**Figure 3:**
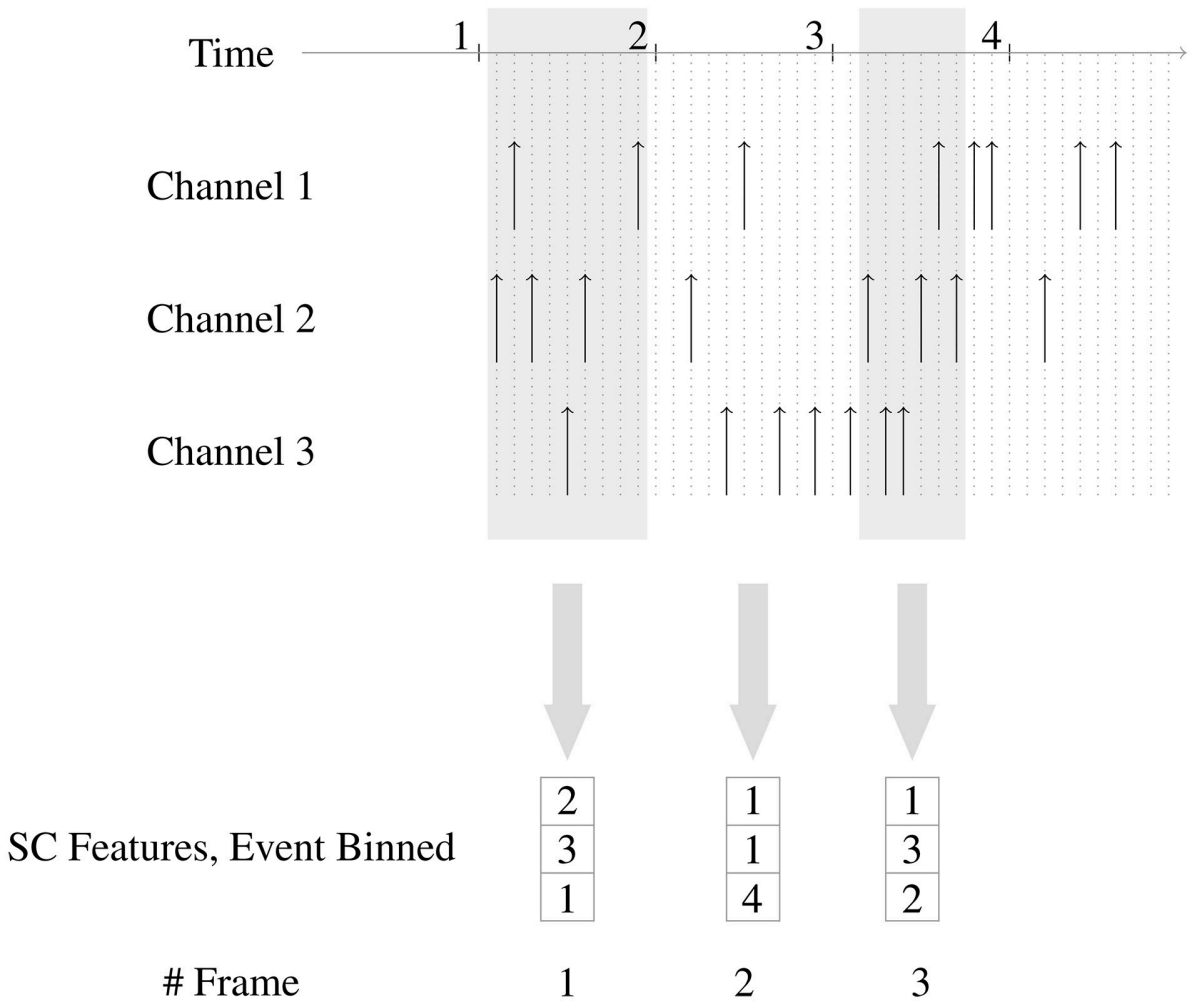
Generation of event-binned Spike Count features. Three channels are shown in this example. Every time window frame used for binning the events has 6 events and there is no overlap of events between consecutive time window frames. In the second time frame, the 6 events are distributed as 1, 1, and 4 across channels 1, 2, and 3, respectively, hence the corresponding feature is (1, 1, 4).

Figure 3 shows how the event-binned spike count features are generated from the spikes.

#### 2.2.4. Comparison of time binning and event binning

**Figure 4:**
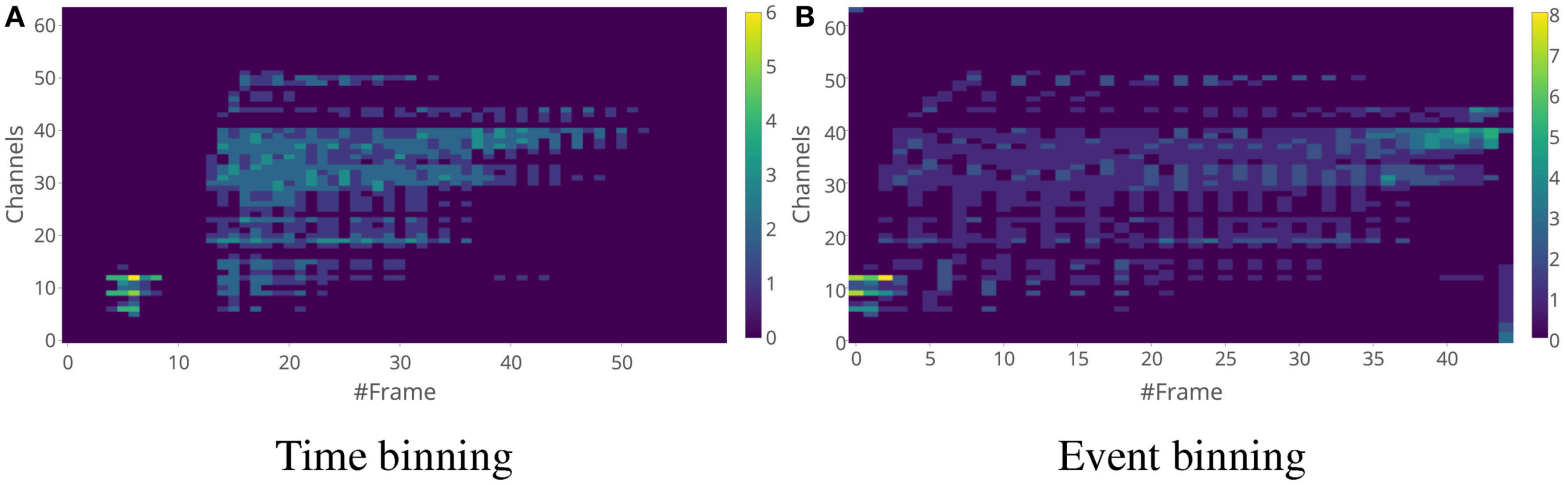
Spike Count features for a digit sample “2”. The time window length for time binning in **(A)** is 5 ms and the number of events in a single frame for event binning in **(B)** is 25. There does not seem to be a clear advantage of choosing event binning over time binning when it comes to individual digits. Note that event binning for this example produces fewer frames compared to the time binning.

**Figure 5:**
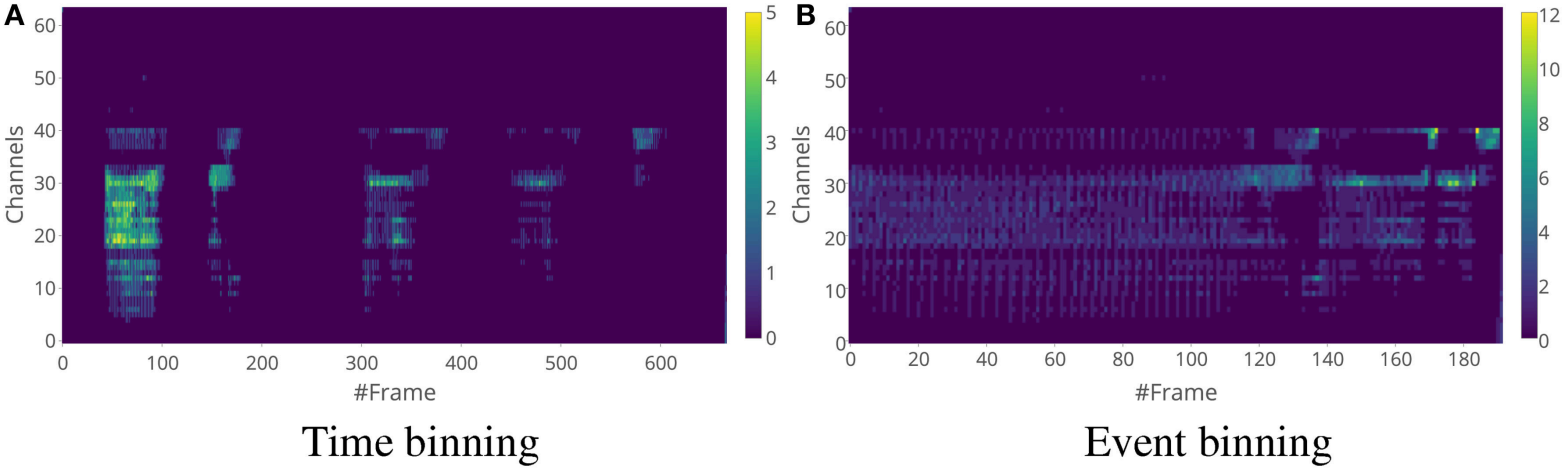
Spike Count features for a digit sequence “5-8-9-9-2”. The time window length for time binning in **(A)** is 5 ms and the number of events in a single frame for event binning in **(B)** is 25. The event binning method does not completely encode the timing information in the sample. Also, the silence periods between the digits is absent in the event-binned features.

Although both methods capture the distribution of the events across the frequency channels, there is a difference between the features generated from these methods. The main difference is that the time window used for time binning is of constant length, while the time window of the event-binned features are of varying lengths. The lengths depend on the input event rate over time. This can be seen in the examples of time-binned and event-binned SC features for a single word as shown in Figure 4 and for a sentence as shown in Figure 5. In Figure 5, it can be seen that the information about silences in the sentence is temporally smeared in the event-binned features. This property is not desirable as it could be a disadvantage when trying to extract information that depend on the silence periods within the sentences, unless silence segmentation is done before generating the features.

#### 2.2.5. Data-driven time-binned spike count features

Further, a data-driven time-binning method is introduced and employed in this work. In contrast to the previous time-binned SC features described in section 2.2.2, a feature frame is not processed if no spikes occurred within the corresponding time bin. In addition, this method specifically uses a brief time-bin length. This allows fewer inputs compared to time-binned spike counts (as a fixed-size vector is either presented or skipped), and far fewer inputs to be presented to the network compared to sequentially presenting raw events while maintaining much of the time resolution. Here, using a short time-bin length allows a high degree of spike time accuracy to be maintained, as individual spikes have correct timestamps discretized to the bin length. These data-driven time-binned SC features *F*^*d*^ can be defined as

(4) $$\begin{array}{l} F_{j}^{d}=F_{i}^{tb},\text{where }i\text{ is such that }\text{max }\left(F_{i}^{tb}\right)>0\text{ and}\\ \;\text{card}\left(\left\{k|k\leq i,\text{max}\left(F_{k}^{tb}\right)>0\right\}\right)=j \end{array}$$

#### 2.2.6. Exponential features

Finally, we introduce a real-valued feature representation that is more amenable to training deep neural networks. This feature is created by convolving each spike with an exponential kernel, that captures the timing information carried by the spikes and has been used in various models, for e.g., Abdollahi and Liu (2011) and Lagorce et al. (2015, 2016). Exponentials are frequently used in neuronal models such as the exponential integrate-and-fire model (Brette and Gerstner, 2005). Although other kernels such as the Gaussian kernels used in the analysis of neuronal firing patterns (Szűcs, 1998) can also be used, we restrict our study here to exponential kernels because they can be applied easier to create real-time features. The resulting output after the convolution is sometimes treated as a real-valued time surface as described in Lagorce et al. (2016). These exponential features have also been used in classification tasks such as image classification (Tapson et al., 2013; Cohen et al., 2016). We first describe the creation of the exponential features and then the binning methods used on these features.

For an audio event stream defined as in Equation (1), the exponential feature $F_{i}^{e}$ for an event *e*_*i*_ is constructed by first defining a time context $T_{i}$ for the event. The time context is an *N*_*c*_ dimensional vector where *N*_*c*_ is the number of frequency channels in the audio sensor and is defined as

(5) $$\begin{array}{l} T_{i}\left(f\right)=\underset{j\leq i}{\text{max }}\left\{t_{j}|f_{j}=f\right\} \end{array}$$

where *f* is the position of a frequency channel. The exponential feature for an event is then defined as

(6) $$\begin{array}{l} F_{i}^{e}\left(f\right)=e^{-\left(t_{i}-T_{i}\left(f\right)\right)/\tau } \end{array}$$

**Figure 6:**
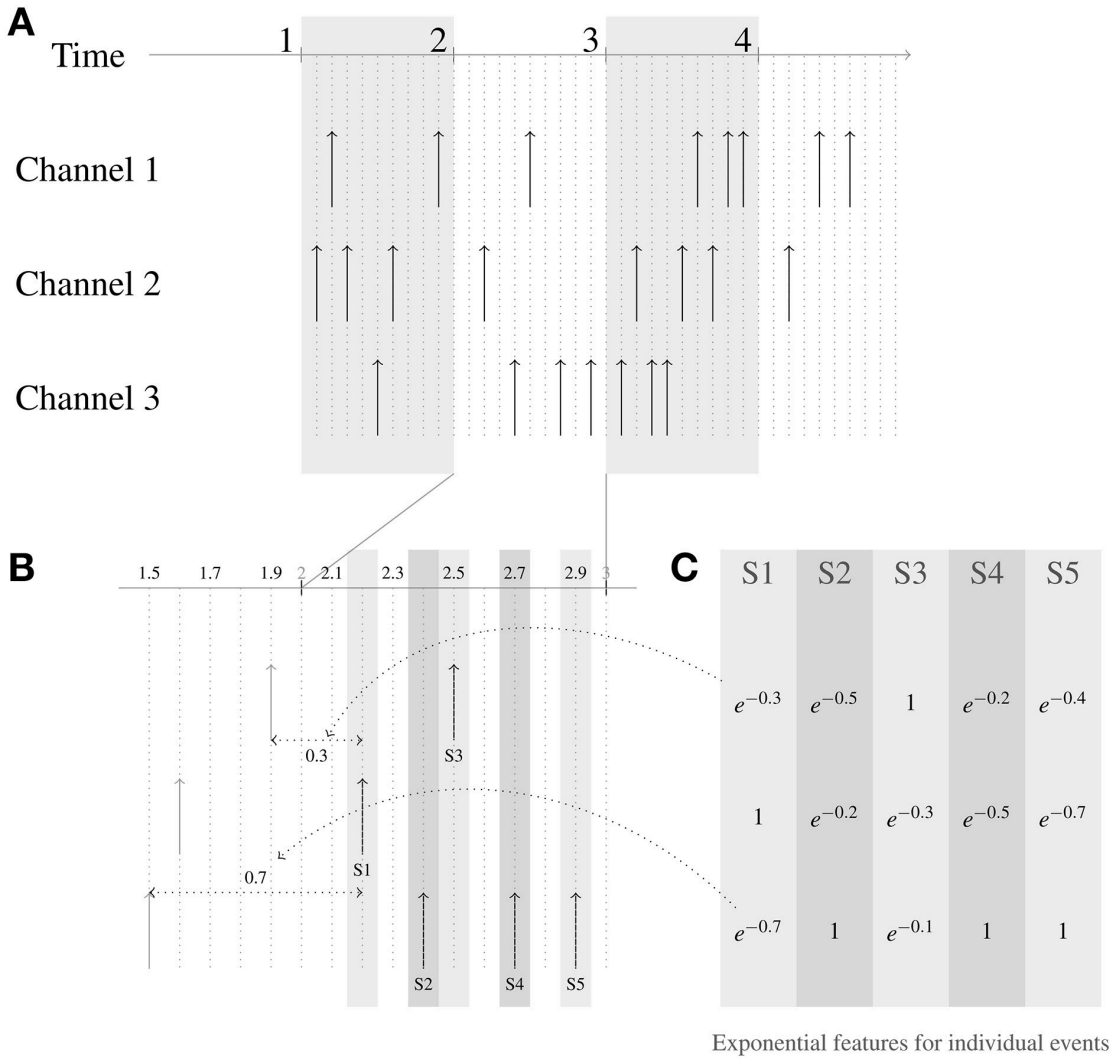
Generation of exponential features for events. Three channels are shown in this example. The time constant parameter t used for generating the features is 1 time unit. The events streams are shown in **(A)**, the zoomed-in picture of the events in the second frame are shown in **(B)**, and the exponential features for this frame is shown in **(C)**. Consider the event at time *t* = 2.2, labeled S1. In channel 1, the closest event in time to the current event occurred 0.3 time units before, and thus the corresponding feature value for the channel 1 in the exponential feature vector for event S1 is *e*^−(0.3/1)^. Similarly for channel 3, the closest event in time to the current event occurred 0.7 time units before, and thus the corresponding entry for channel 3 in the exponential feature for S1 is *e*^−(0.7/1)^. For channel 2, since the current event is at channel 2, the exponential feature value at channel 2 is *e*^−(0/1)=1^.

An illustration describing the generation of the exponential features for the events is shown in Figure 6.

**Figure 7:**
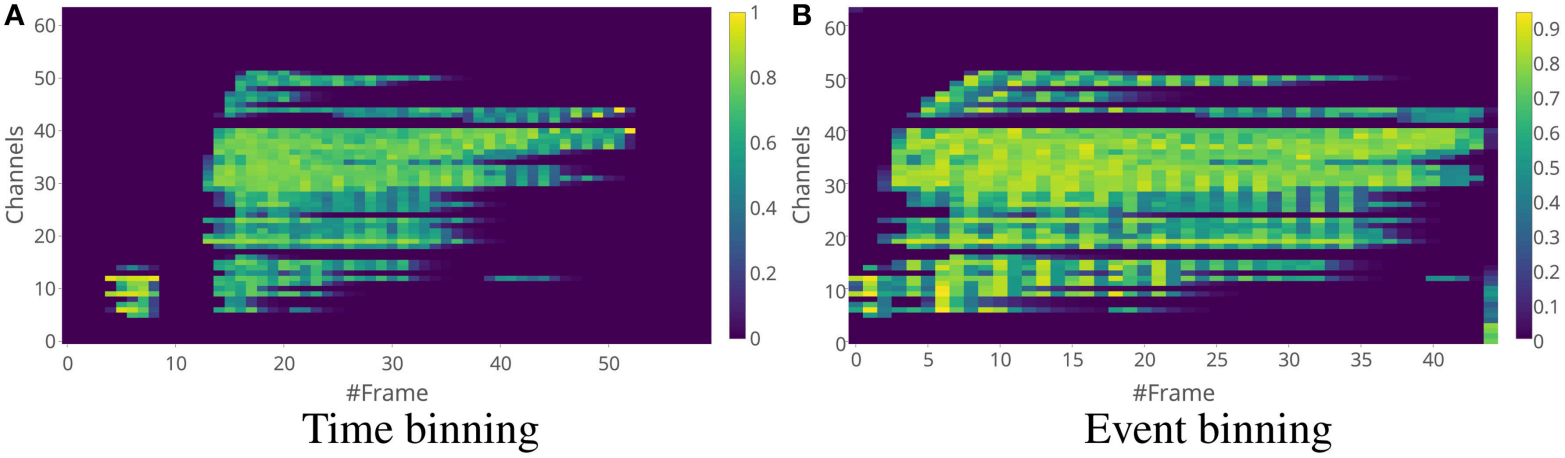
Exponential feature examples for the same word as in Figure 4. The time window length for time binning in **(A)** is 5 ms and the number of events in a single frame for event binning in **(B)** is 25. One main difference between the spike count features and the exponential features is that the exponential feature values are in the range between 0 and 1, while the spike count feature values depend on the volume of the spikes in the time window.

**Figure 8:**
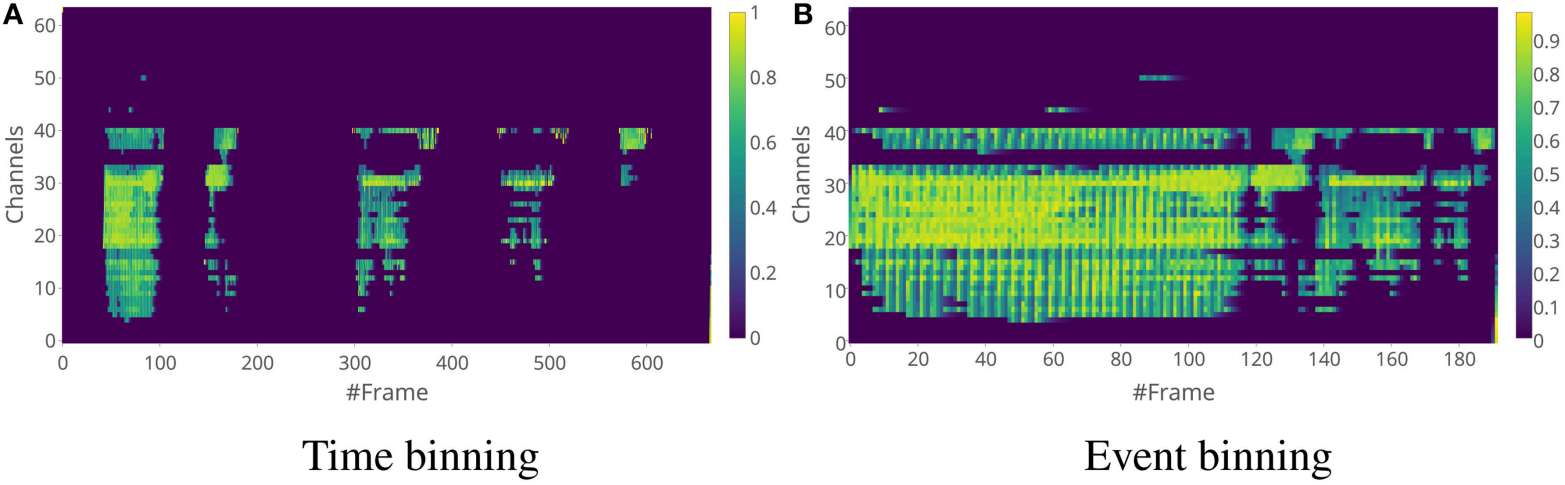
Exponential feature examples for the same sequence as in Figure 5. The window length for time binning in **(A)** is 5 ms and the number of events in a single frame for event binning in **(B)** is 25.

Once these exponential features are created, the events are binned into time window frames either through time binning or event binning like in the SC features, and the average of the exponential features for the events in the time window frame is used as the exponential feature for the frame. For the rest of the paper, we use the term “exponential features" to mean exponential features for a frame. Examples of time binning and event binning exponential features for a single word are shown in Figure 7 and for a sentence are shown in Figure 8.

For a real-time implementation, the exponential features are computed recursively as follows.

(7) $$\begin{array}{l} F_{i}^{e}\left(f\right)=\left\{ \begin{array}{cc} e^{-\left(t_{i}-t_{i-1}\right)/\tau }F_{i-1}^{e}\left(f\right),\; & \text{if}f\neq f_{i}\\ 1,\; & \text{if}f=f_{i} \end{array}\right. \end{array}$$

With $F_{0}^{e}$ initialized to a zero vector, it can easily be seen that the above implementation corresponds to the definition in Equation (6).

### 2.3. Recurrent neural networks

Convolutional Neural Networks are typically used in vision classification tasks and have been successfully used together with the Dynamic Vision Sensor (Moeys et al., 2016). These networks have a feedforward architecture where the neurons in one layer only drive the neurons in the upper layers. However, recurrent neural networks (RNNs) in which neurons in one layer recurrently receive input from neurons in the same layer, are more generally used when the inputs consist of temporal sequences.

Given a sequence *x* = (*x*_1_, *x*_2_, …, *x*_*T*_), the RNN layer updates its hidden state *h*_*t*_ with *t* ∈ {0, 1, 2, …, *T*}, with *h*_0_ being the initial state and *h*_*t*_ = ϕ(*h*_*t*−1_, *x*_*t*_), where ϕ is a non-linear function. Generally, the update function for the hidden state is of the form *h*_*t*_ = φ(*Uh*_*t*−1_ + *Wx*_*t*_), where *U* and *W* are connection matrices of appropriate sizes and φ is an activation function such as a logistic sigmoid or the hyperbolic tangent (Chung et al., 2014).

Training RNNs using gradient descent to learn long term time dependencies in the input is difficult because of the vanishing/exploding gradient problem (Bengio et al., 1994). In order to counter this problem, the Long-Short Term Memory (LSTM) (Hochreiter and Schmidhuber, 1997) neuron model was proposed. This model has an activation function that is managed by different gates acting like a memory control for the neuron. The subsequently proposed Gated Recurrent Unit (GRU) (Cho et al., 2014) model performs well on similar tasks and has the advantage of using fewer parameters. In our experiments, we use both GRU and LSTM RNNs and the following sections introduce these models.

#### 2.3.1. Long-short term memory

The form of LSTM used in this work derives from Graves (2013):

(8) $$\begin{array}{l} i_{t}=\sigma_{i}\left(W_{xi}x_{t}+W_{hi}h_{t-1}+w_{ci}⊙c_{t-1}+b_{i}\right) \end{array}$$

(9) $$\begin{array}{l} f_{t}=\sigma_{f}\left(W_{xf}x_{t}+W_{hf}h_{t-1}+w_{cf}⊙c_{t-1}+b_{f}\right) \end{array}$$

(10) $$\begin{array}{l} c_{t}=f_{t}⊙c_{t-1}+i_{t}⊙\sigma_{c}\left(W_{xc}x_{t}+W_{hc}h_{t-1}+b_{c}\right) \end{array}$$

(11) $$\begin{array}{l} o_{t}=\sigma_{o}\left(W_{xo}x_{t}+W_{ho}h_{t-1}+w_{co}⊙c_{t}+b_{o}\right) \end{array}$$

(12) $$\begin{array}{l} h_{t}=o_{t}⊙\sigma_{h}\left(c_{t}\right) \end{array}$$

The introduction of gating functions in Hochreiter and Schmidhuber (1997) differed from traditional RNNs, and allowed substantially easier training for recurrent networks. The gate activation vectors, *i*_*t*_, *f*_*t*_, *o*_*t*_, represent the input, forget, and output gates respectively. Each neuron stores an internal cell activation vector *c*_*t*_, while the input and hidden state vectors are *x*_*t*_ and *h*_*t*_, respectively. A sigmoidal nonlinearity, *y* = 1/(1+*e*^−*x*^), is applied to constrain the gates to lie between 0 and 1, and applied to the gates with σ_*i*_, σ_*f*_, and σ_*o*_ for the input, forget, and output gates. For these gates, each gate has a weight parameter for the input *x* and the hidden state *h*, resulting *W*_*xi*_ and *W*_*hi*_, *W*_*xf*_ and *W*_*hf*_, *W*_*xo*_ and *W*_*ho*_ for the input, forget, and hidden gates, respectively. Additionally, each gate has a bias *b*_*i*_, *b*_*f*_, and *b*_*o*_ for the input, forget, and output gates. The ⊙ notation signifies an elementwise (Hadamard) product, implying that each cell state *c*_*t*_ is a linear interpolation between the previous cell state (controlled by *f*_*t*_) and the new cell state (controlled by *i*_*t*_). Finally, the cell state is transformed by the output gate *o*_*t*_ to produce a new hidden state *h*_*t*_. Optionally, peephole connections, Gers and Schmidhuber (2000) *w*_*ci*_, *w*_*cf*_, and *w*_*co*_, are commonly employed for the cell state *c*_*t*_ to further influence the input, forget, and output gates.

#### 2.3.2. Gated recurrent units

Another commonly used gated architecture is the GRU architecture. The primary difference compared to LSTM is the removal of one gate, which results in faster training and execution time while achieving approximately the same accuracy in most tasks. The form employed in this work is the most common implementation from Chung et al. (2014):

(13) $$\begin{array}{l} r_{t}=\sigma_{r}\left(W_{xr}x_{t}+W_{hr}h_{t-1}+b_{r}\right) \end{array}$$

(14) $$\begin{array}{l} u_{t}=\sigma_{u}\left(W_{xu}x_{t}+W_{hu}h_{t-1}+b_{u}\right) \end{array}$$

(15) $$\begin{array}{l} c_{t}=\sigma_{c}\left(W_{xc}x_{t}+r_{t}⊙\left(W_{hc}h_{t-1}\right)+b_{c}\right) \end{array}$$

(16) $$\begin{array}{l} h_{t}=\left(1-u_{t}\right)⊙h_{t-1}+u_{t}⊙c_{t} \end{array}$$

Similar to the above, there are the gate states *r*_*t*_ and *u*_*t*_, referred to as the reset and update gates, as well as a combination gate and state *c*_*t*_ called the candidate state. As above, each consists of the application of a matrix multiplication of a weight vector (*W*_*xr*_, *W*_*xu*_, *W*_*xc*_) to the input (*x*_*t*_), as well as another matrix multiplication of a weight vector (*W*_*hr*_, *W*_*hu*_, *W*_*xc*_) to the previous hidden state (*h*_*t*−1_), for the reset, update, and candidate gates respectively. For the two pure gates, these two terms are summed with a bias (*b*_*r*_ and *b*_*u*_) and the logistic sigmoid nonlinearity *y* = 1/(1 + *e*^−*x*^) is applied to constrain each gate to lie between 0 and 1. For the candidate state, the reset gate is applied elementwise to the previous hidden state, and the bias *b*_*c*_ is added before the candidate state is transformed nonlinearly using the same logistic sigmoid. Finally, the new hidden state *h*_*t*_ is the result of a linear mixture of the update gate elementwise applied to the candidate state, and the previous hidden state controlled by the complement of the update gate.

#### 2.3.3. Phased LSTM

The Phased LSTM model, which was introduced in Neil et al. (2016), equips the LSTM model with the ability to process irregularly-sampled continuous-time sequences through the application of a novel time gate *k*_*t*_. This time gate, similar to other gates, produces a continuous value between 0 and 1 but is instead controlled by an external timing input. Each neuron has independent, learnable timing parameters that allow the neuron's time gate to execute a rhythmic wake-sleep cycle over time. When the time gate is open (close to 1), the neuron performs as a normal LSTM neuron does; when the time gate closes (close to 0), the neuron performs no updates until its next wake period. Other neurons, however, can still inspect a sleeping neuron's state. When continuous time sequences are applied, the timestamp of the event controls which subset of neurons are updated, and permits calculations based on the rhythmic wake-sleep cycle of the neurons in response timestamp itself.

Rigorously, the opening and closing of the gate is a periodic oscillation controlled by three parameters: a period τ that controls the duration, a shift *s* that applies a phase shift offset, and an on ratio *r*_*on*_ that determines the duration of the open period. The time (khronos) gate *k*_*t*_ can be calculated as:

(17) $$\begin{array}{l} \phi_{i,t}=\frac{(t-s_{i})\mathrm{mod}\tau_{i}}{\tau_{i}},\;k_{i,t}=\;\{\begin{array}{ll} \frac{2\phi_{i,t}}{r_{on,i}}, & \text{if }\phi_{i,t}<\frac{1}{2}r_{on,i}\\ 2-\frac{2\phi_{i,t}}{r_{on,i}},\; & \text{if}\frac{1}{2}r_{on,i}<\phi_{i,t}<r_{on,i}\\ \alpha \phi_{i,t}, & \text{otherwise} \end{array} \end{array}$$

The neuron index *i* indicates which parameters are neuron-specific (ϕ_*i,t*_, *k*_*i,t*_, *s*_*i*_, τ_*i*_, *r*_*on,i*_) and which are global (*t*, α). Here, ϕ_*i,t*_ is introduced as an auxiliary variable to represent the percentage of the phase within the rhythmic cycle, ranging from 0 to 100%. There are three piecewise phases of the operation of the gate functionally represented in Equation (17): an open and rising phase (during the first half of *r*_*on*_), an open and falling phase (during the second half of *r*_*on*_) and an off phase. The linear slopes of the rising and falling phase have a constant gradient to preserve strong gradient information, in the same manner that allows ReLUs to train so well (LeCun et al., 2015). Further, note a leak is applied during the off phase with analogy to the leaky rectified linear unit (He et al., 2015) to permit the flow of gradient information even when the neuron is off. However, after training, the leak can be set to zero (i.e., α = 0) and thus truly off, so no updates need be performed when the neuron is in the closed or sleep phase of the cycle. This continuous-time equation is defined at all time points *t* but requires no computation between sampled data points, allowing irregularly-spaced points in time to be effectively used within this framework as the neurons have an explicit model of time. The LSTM equations from above can then be rewritten to permit arbitrary time points *j* rather than timestep indices, using a proposed cell state $\tilde{c_{j}}$ and proposed hidden state $\tilde{h_{j}}$ controlled by the time gate *k*_*j*_:

(18) $$\begin{array}{l} i_{j}=\;\sigma_{i}\left(W_{xi}x_{j}+W_{hi}h_{j-1}+w_{ci}⊙c_{j-1}+b_{i}\right) \end{array}$$

(19) $$\begin{array}{l} f_{j}=\;\sigma_{f}\left(W_{xf}x_{j}+W_{hf}h_{j-1}+w_{cf}⊙c_{j-1}+b_{f}\right) \end{array}$$

(20) $$\begin{array}{l} \tilde{c_{j}}=\;f_{j}⊙c_{j-1}+i_{j}⊙\sigma_{c}\left(W_{xc}x_{j}+W_{hc}h_{j-1}+b_{c}\right),\\ \;c_{j}=k_{j}⊙\tilde{c_{j}}+\left(1-k_{j}\right)⊙c_{j-1} \end{array}$$

(21) $$\begin{array}{l} o_{j}=\;\sigma_{o}\left(W_{xo}x_{t}+W_{ho}+w_{co}⊙\tilde{c_{j}}+b_{o}\right) \end{array}$$

(22) $$\begin{array}{l} \tilde{h_{j}}=\;o_{j}⊙\sigma_{h}\left(\tilde{c_{j}}\right),\\ \;h_{j}=k_{j}⊙\tilde{h_{j}}+\left(1-k_{j}\right)⊙h_{j-1} \end{array}$$

The sparseness in time of computation (typically, with *r*_*on*_ set to 5%) allows this implementation to be far sparser than traditional gated implementations in computation while maintaining high performance. Furthermore, as timesteps are no longer required and the neuron has an explicit model of time, even raw spike events can be directly used with Phased LSTM. For further information, refer to the formulation of Phased LSTM in Neil et al. (2016) or one of its publicly-available implementations^1^^,^^2^.

### 2.4. Datasets

This paper introduces the N-TIDIGITS18 dataset by playing the audio files from the TIDIGITS dataset to the CochleaAMS1b sensor. The dataset is publicly accessible at http://sensors.ini.uzh.ch/databases.html. The dataset includes both single digits and connected digit sequences, with a vocabulary consisting of 11 digits (“oh,” “zero” and the digits “1” to “9”). Each digit sequence is of length 1–7 spoken digits. There is a total of 55 male and 56 female speakers in the training set with a total of 8,623 training samples, while the testing set has a total of 56 male and 53 female speakers with a total of 8,700 testing samples.

The entire dataset is used or a reduced version of the dataset is used where only the single digit samples are used for training and testing. In the single digits dataset, there are two samples for each of the 11 single digits from every speaker, with a total of 2,464 training samples and 2,486 testing samples. The N-TIDIGITS18 dataset with all the samples was used to train a sequence classification task while the digit samples were used to train a digit recognition task. For most of our training, unless specified, we only use events from one ear and one neuron.

### 2.5. Network architectures and training criterion

#### 2.5.1. GRU/LSTM architectures

Two network models were trained separately for the digit recognition task and the sequence classification task. For the digit recognition task, the network consists of 2 GRU layers with 100 units each, followed by a fully connected layer of 100 units with a ReLU activation followed by a Softmax classification layer. For the sequence classification task, each network consists of a fully connected layer of 100 units with SELU activation (Klambauer et al., 2017) followed by 2 LSTM layers of 100 units each followed by the final classification layer. The recently introduced SELU activation helps with regularization of the network by pushing the neuronal activations of the corresponding layer to zero mean and unit variance without the need for batch normalization. The SELU activation function was used over other activation functions mainly because the overall accuracy was significantly improved by using it.

The network for the digit recognition task was trained using a categorical cross entropy objective, while the network for the sequence classification task was trained using the Connectionist Temporal Classification (CTC) objective (Graves et al., 2006). For the CTC objective on sequence classification, the accuracy metrics used were the label error rate and the phrase error rate. For the label error rate, we first calculate the average edit distance (Levenshtein, 1966) between the correct label sequences and the corresponding predicted label sequences. The edit distance between two sequences is the minimum number of insertions, deletions and substitutions required to transform one into the other. The label error is then given by the ratio of the calculated average edit distance and the total number of labels in the correct label sequences. This metric is not a strict proper fraction for its value can go above 1. The phrase error rate is given by the ratio of the correctly predicted label sequences and the total number of label sequences.

All networks were trained on the Tensorflow framework (Abadi et al., 2015) using Adam optimizer with a learning rate of 0.001 over 200 epochs. All the presented accuracy numbers are based on at least three experimental runs. The network and simulation parameters are summarized in Table 1.

**Table 1:** Summary of the different training parameters used in this study.

**Network**	**Model architecture**	**Batch size**	**No.of epochs**
GRU RNN	2x 100 GRU - 100 Dense (ReLU) - 10 Softmax	128	200
LSTM RNN	100 Dense (SELU) - 2x 100 LSTM - 10 Dense	128	200
Phased LSTM	2x 250 Phased LSTM - 10 Dense	16	50

*Tha Adam optimizer with a learning of 0.001 was used for all the networks*.

#### 2.5.2. Phased LSTM architecture

The single-event architecture was used on the raw input spikes. Because of the volume of input spikes and the difficulty in training extremely long sequences, only one neuron (out of four possible neurons) from one ear was used, resulting in sequences of approximately 4,000 spikes. Because a raw spike address and the corresponding spike time was used, an embedding layer of size 40 was used. As in Neil et al. (2016), a multi-resolution embedding layer downsamples the address by 1, 2, 4, and 16, and concatenates the 10-dimensional embedded feature from each result together. This allows learning features across multiple pitches (neuron addresses) as well as learning features particular to each pitch. After the embedding layer, two layers of 250 Phased LSTM neurons are included, with period τ~exp(U(0,3)) milliseconds (with x~U(a,b) implying a random draw of x from the uniform distribution between limits *a* and *b*), shifts s~U(0,100) milliseconds, and the on ratio *r*_*on*_ = 0.05 resulting in 5% activity. The output of the second Phased LSTM layer is fully connected via a dense layer to the ten output classes.

For the N-TIDIGITS18 dataset, only 40% of 0.5 ms time bins (also timesteps) have data (with an average of 3.6 spikes per time bin), running at a 2.5× increase in speed over calculating every timestep. Furthermore, the number of bins are far fewer in number than the number of input spikes as would be the case with processing the raw input data. Compared to processing every spike sequentially in the full dataset (all neurons, all ears), there are now 30 times fewer timesteps, resulting in a dramatic decrease in training time when training on data-driven bins.

All Phased LSTM networks were trained on the Lasagne framework (Dieleman et al., 2015) using the Adam optimizer and a learning rate of 0.001 over 50 epochs.

## 3. Results

We present the network accuracy results of the different pre–processing methods on the audio classification tasks based on the N-TIDIGITS18 dataset when these features are presented to the different recurrent models.

### 3.1. Comparison of feature representations

The performance of the pre–processed features are tested through two classification tasks. The first is a word recognition task on the single digit samples in the dataset, and the second is a sentence prediction task on the connected digit samples. The classifiers used for different tasks and their performances on the different feature types are shown in Table 2. The results in the table show that the networks using the exponential features consistently perform better than the spike count features across both the tasks. The Phased LSTM networks which were used to process either the raw event data or the data-driven bins outperform the spike count features, and produce similar accuracies as GRU RNNs with exponential features.

**Table 2:** Summary of investigated models on N-TIDIGITS18 dataset.

**Feature type**	**Sensor**	**Task**	**Classifier**	**Accuracy (%)**
**MFCC**		**Digit**	**GRU RNN**	**97.90**
Binned frames (fixed bins/sample)^*^	AMS1b	Digit	SVM	95.08
Constant time bins^**^	AMS1b	Digit	CNN	87.65
Constant time bins^**^	AMS1b	Digit	GRU RNN	82.82
Single events (raw data)	AMS1b	Digit	Phased LSTM	87.75
Data-driven time-binned features	AMS1b	Digit	Phased LSTM	91.25^a^
Constant time bins	AMS1b	Digit	GRU RNN	86.4
**Exponential features**	**AMS1b**	**Digit**	**GRU RNN**	**90.9**
Constant time bins	AMS1c	Digit	GRU RNN	88.6
**Exponential features**	**AMS1c**	**Digit**	**GRU RNN**	**91.1**
Constant time bins	AMS1b	Sequence	LSTM RNN	86.1^b^
**Exponential features**	**AMS1b**	**Sequence**	**LSTM RNN**	**87.3**^b^

*The MFCC features are extracted from the original TIDIGITS dataset*.

a *Events from all neurons and both ears used in training*.

b *Label accuracy on sequences*.

* *Abdollahi and Liu (2011)*.

** *Neil and Liu (2016)*.

Although the Phased LSTM network takes a longer time to train because the input sequences of single spikes are longer, one advantage of this method over the other pre-processing methods is that there are no hyper parameters that need fine tuning such as the time window length parameter *T*_*l*_ used for binning or the tau parameter τ used in the exponential features.

The performance of the method using the binned frames on a Support Vector Machine (SVM) classifier is better than all the other methods but this method relies on access to the whole sample which is then converted into a fixed number of bins per sample and unfortunately cannot be used on a real-time recognition system.

### 3.2. Optimizing parameters

As discussed in section 3.1, both spike count features and exponential features have a few hyperparameters that need fine tuning for optimal performance. The network hyperparameters were optimized once using a small validation split on the training data from the N-TIDIGITS18 dataset. The small validation dataset was created by using 10% of the training samples while the model was trained on the other 90% of the samples.

**Figure 9:**
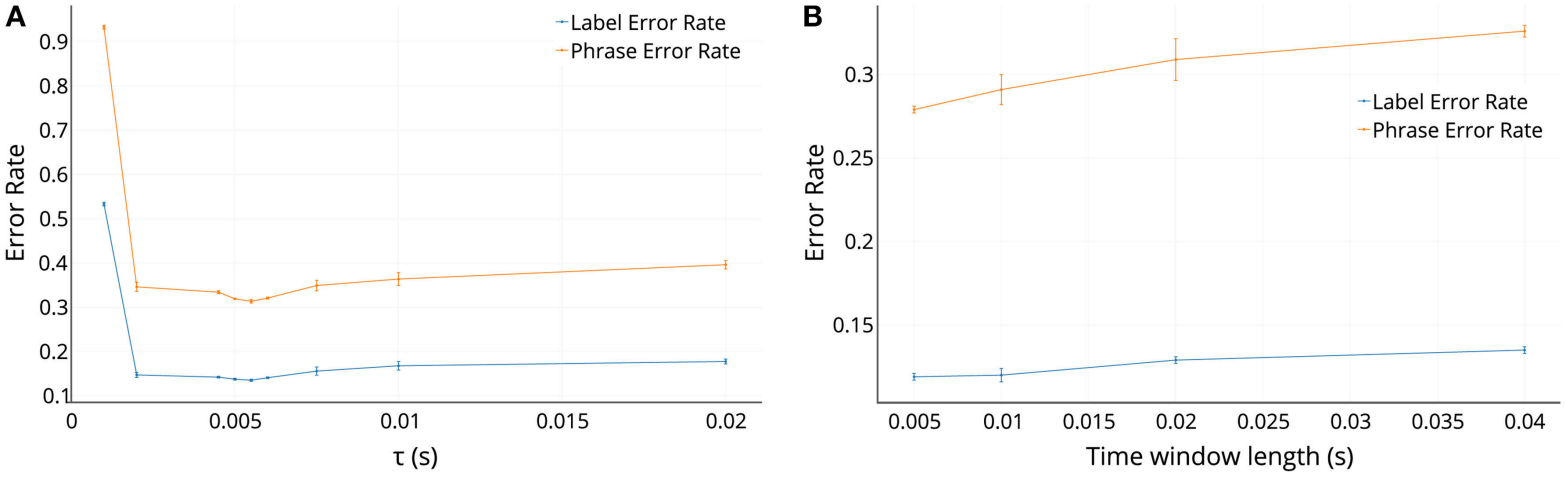
The effect of τ and time window length parameters on the error rates in the sequence classification task in the case of exponential features. The window length for time binning in **(A)** is 5 ms and the value of τ used in **(B)** is 5.5 ms. Although using the optimal values gives lower error rates, using a larger time window length than the optimal value of 5 ms would help in having shorter sequences to process.

The variation of the error rates on the τ parameter and the *T*_*l*_ parameter for the exponential features in the sequence classification task are shown in Figures 9A,B, respectively. In Figure 9A, we can see that the error rate is very high for τ less than 2 ms, and then remains fairly steady for values of τ till 5 ms and then rises slowly as τ increases. The optimal value of τ is related to the mean inter-spike interval in the frequency channels. While for low values of τ the features do not properly encode the history of the events and thus do not perform well, while for increasing τ the exponential feature values saturate toward 1 and thus do not provide enough contrast among the features for the classification tasks to decode properly.

In Figure 9B, we can see that the error rates increase with larger *T*_*l*_, because with a bigger *T*_*l*_, the exponential features get smeared because of averaging over more events. But it should be considered that with increasing *T*_*l*_, the sequences to be processed become shorter which makes the recurrent network training easier and efficient.

These plots suggest that the optimal τ is 5.5 ms and the optimal *T*_*l*_ is 5 ms, which were also the values used in the experiments for Table 2. Although the optimal value of *T*_*l*_ was 5 ms, using a parameter value of 40 ms would help save training time and number of computes required to process the network per unit time since there are fewer frames to process per unit time. This advantage comes though at the expense of a reduced accuracy of about 1.6% (88.1% for 5 ms and 86.5% for 40 ms) on the validation set.

### 3.3. Comparison of processing times for feature generation

To compare the processing times of conventional MFCC with the SC and exponential filters, we used a Raspberry Pi 3 Model B, with ARM Cortex A-53 processor. We used a feature frame rate of 100 Hz. We created random data for both the raw audio and the event data. For raw audio we used a sampling rate of 16 kHz with uniformly sampled data between [−1, 1] at every sampling point. Since the observed average spike rate was about 3,400 Hz for the N-TIDIGITS18 dataset, for the computational test with event data, we generated Poisson spike trains with a total spike rate of 4,000 Hz. Across 100,000 runs, the average processing times per frame on the hardware were 5.79 ms for the MFCC features, 0.72 ms for the SC features and 2.2 ms for the exponential features. Thus the event-driven features are faster to generate by a factor of 2.2X for exponential features and 8X for SC features. This result is not surprising given the computational simplicity of the cochlea features afforded by the sensor preprocessing.

## 4. Discussion

In this work, we performed a comparative study of the performance accuracy of a gated recurrent neural network that processes either the raw audio spikes or framed features extracted by different spike processing methods. We demonstrate the use of a recent LSTM model called the Phased LSTM which operates on raw audio spikes. We compared the performance accuracy of this model to that of the standard gated recurrent neural networks, the LSTM and the GRU networks, that processed framed features extracted by different spike processing methods.

The results show that it is possible to achieve a good performance through processing the raw events using the Phased LSTM model. This model, designed for use on long sequences, makes use of the inherent timing information present in the spikes. Although the training time is long because the model has to learn to process more timesteps, there are no meta-parameters to tune in the feature generation.

Alternatively, pre-processing the spikes to produce framed features is appealing because the input sequences to the recurrent networks will be shorter than the sequence of raw events. For both the single digit and digit sequence datasets, the network classification accuracy is higher by approximately 2.5% when using exponential features over spike count features. It should be noted that the results are obtained on the N-TIDIGITS18 dataset, a relatively small dataset. We will investigate in the future if the higher accuracy from using exponential features extend to larger datasets.

We hypothesize that the increased accuracy from exponential features is due to two reasons. First, interspike intervals in the spike streams carry information useful for the classification task and therefore exponential features are more desirable. Second, the encoded exponential feature values are real-valued and range between 0 and 1 while the spike count feature values are quantized in discrete quantities of 1. Having real-valued input features might help during training of the recurrent networks.

Even though the accuracy results from using the pre-processed audio spike frames were lower than the results obtained from using MFCC features, the focus of this work is to present improved methods for processing the outputs of event driven sensors in real-time applications. Our evaluation of the average processing time per frame on a Raspberry Pi shows that generation of the event-driven features is faster than that of MFCCs by a factor of 2–8 depending on the cochlea features used. We also aim towards an event-driven system where processing would be activated only if there are sufficient spikes from the sensor, e.g., the processing is inactivated during silent periods.

The results presented here serve as a baseline for future studies on algorithms that process spikes from spiking audio sensors. The pre-processing methods and the LSTM/GRU networks used in the work above are already implemented in real time (Anumula et al., 2017) using the jAER framework (Delbruck, 2008). The N-TIDIGITS18 dataset used in our experiments is publicly accessible at http://sensors.ini.uzh.ch/databases.html.

## Author contributions

JA performed the RNN experiments and contributed to the writing, DN performed the Phased LSTM experiments and contributed to the writing, TD contributed to discussions on the feature extraction methods and assisted in the development of the hardware infrastructure of the cochlea boards, and S-CL contributed to the design of the experiments and the writing.

### Conflict of interest statement

The authors declare that the research was conducted in the absence of any commercial or financial relationships that could be construed as a potential conflict of interest.

## Footnotes

^1^https://github.com/dannyneil/public_plstm

^2^https://www.tensorflow.org/api_docs/python/tf/contrib/rnn/PhasedLSTMCell
